# Medical Image Segmentation Algorithm Based on Feedback Mechanism CNN

**DOI:** 10.1155/2019/6134942

**Published:** 2019-08-01

**Authors:** Feng-Ping An, Zhi-Wen Liu

**Affiliations:** ^1^School of Physics and Electronic Electrical Engineering, Huaiyin Normal University, Huaian JS 223300, China; ^2^School of Information and Electronics, Beijing Institute of Technology, Beijing BJ 100081, China

## Abstract

With the development of computer vision and image segmentation technology, medical image segmentation and recognition technology has become an important part of computer-aided diagnosis. The traditional image segmentation method relies on artificial means to extract and select information such as edges, colors, and textures in the image. It not only consumes considerable energy resources and people's time but also requires certain expertise to obtain useful feature information, which no longer meets the practical application requirements of medical image segmentation and recognition. As an efficient image segmentation method, convolutional neural networks (CNNs) have been widely promoted and applied in the field of medical image segmentation. However, CNNs that rely on simple feedforward methods have not met the actual needs of the rapid development of the medical field. Thus, this paper is inspired by the feedback mechanism of the human visual cortex, and an effective feedback mechanism calculation model and operation framework is proposed, and the feedback optimization problem is presented. A new feedback convolutional neural network algorithm based on neuron screening and neuron visual information recovery is constructed. So, a medical image segmentation algorithm based on a feedback mechanism convolutional neural network is proposed. The basic idea is as follows: The model for obtaining an initial region with the segmented medical image classifies the pixel block samples in the segmented image. Then, the initial results are optimized by threshold segmentation and morphological methods to obtain accurate medical image segmentation results. Experiments show that the proposed segmentation method has not only high segmentation accuracy but also extremely high adaptive segmentation ability for various medical images. The research in this paper provides a new perspective for medical image segmentation research. It is a new attempt to explore more advanced intelligent medical image segmentation methods. It also provides technical approaches and methods for further development and improvement of adaptive medical image segmentation technology.

## 1. Introduction

Medical image enhances the sharpness of the original image by denoising, restoring, etc. or highlights certain information in the image for the segmentation of organs and tissues of interest for quantitative analysis [1–3]. Although there are many kinds of medical image segmentation methods, medical image segmentation methods are mainly divided into the following five categories: medical image segmentation based on thresholds, medical image segmentation based on region growing arithmetic, medical image segmentation based on the deformation model, medical image segmentation based on graph theory, and medical image segmentation based on machine learning [4–6].

The first category is a threshold-based medical image segmentation method. It splits the image based on the difference in grayscale values of the pixels in the image. The method has the advantages of being simple and fast and can achieve efficient segmentation when the image quality is high, the grayscale difference between the target and the background to be segmented is large, and the boundary is clearly distinguished. For example, Wilkins et al. [7] used a threshold method to segment the retinal cystic edema region on the OCT image. However, when the histogram has no obvious troughs, the threshold segmentation method usually does not have a suitable threshold, leading to false segmentation. The threshold segmentation method is sensitive to noise and unevenness. In the segmentation application of medical images, threshold segmentation is usually used as a preprocessing method.

The second category is a medical image segmentation method based on region growing arithmetic. Its basic principle is the process of aggregating image pixels or subregions into larger regions based on user-defined similarity functions. The advantage of this algorithm is that it is simple to calculate and has good accuracy and high efficiency for uniform connected targets. It is sensitive to noise, resulting in a certain void or disconnection in the extracted area. Therefore, it needs to be used in combination with other image processing operations [8, 9].

The third category is a medical image segmentation method based on a deformation model. It comprehensively utilizes regional and boundary information and is the most widely used method. The most typical is the active appearance model (AAM), in which “appearance” [10] refers to outlines and textures, which is an extension of the active shape model. It combines the features of contours and image textures to segment the image more accurately. Mitchell [11] and others used a three-dimensional active appearance model for left and right ventricle segmentation on MR images and achieved good results. The advantages of this method are that it can produce parameter curves or surfaces that are closed in parameters (such as heart contours) or not closed (five description lines in face recognition) and can improve accuracy by using prior knowledge learned in training sets. The disadvantage is that the parameters in the model constrain its flexibility. When the test data are significantly different from the training set data, the accuracy will be greatly reduced.

The fourth category is a medical image segmentation method based on graph theory. It is a new unsupervised image segmentation technique that does not require initialization. Its basic idea is to establish each pixel in the medical image as each node in the graph structure, and they can be abundant. There is also a connection between the foreground and background seed points, and the entire graph is segmented by the method of maximum flow and minimum cut. Both graph cut and graph search methods are effective segmentation methods based on graph theory, and their effectiveness has been proven in images of different modalities [13–15]. One advantage is that the global optimal solution can be obtained according to the designed energy function to avoid falling into a local optimum. One disadvantage is that it is too sensitive to the seed point.

The machine learning-based method can effectively solve the problems that many traditional medical image segmentation methods have had difficulty solving in the past. The main methods are as follows: (1) Medical image segmentation based on boosting: Boosting [12] is a method used to improve the accuracy of learning algorithms by constructing a series of predictive functions and then combining them into a predictive function in a certain way. Freund and Schapire proposed the AdaBoost algorithm; it solved many practical difficulties in the early boosting algorithm. However, it has a weak adaptive ability. (2) Medical image segmentation based on a support vector machine: For example, a deep learning model is used to perform segmentation of MRI images [13] and segmentation of the knee articular cartilage [14]. The support vector machine has a certain effect on medical image segmentation. However, the kernel function selection and weak adaptive ability involved in this method affect its further promotion and application. (3) Medical image segmentation based on neural networks: This method has been promoted and applied in the field of medical image segmentation [15, 16]. However, this kind of method cannot better reflect the characteristics of association and multiscale in the process of medical image segmentation. (4) Medical image segmentation based on deep learning: The convolutional neural network (CNN) was proposed by Lecun et al. It was the first true multilayer structure-learning algorithm that could reduce the number of parameters by using spatial relative relations to improve the training performance [17–19]. It is characterized by the creation and simulation of a neural network for human brain analysis and learning, and it is used to simulate human brain analysis and interpretation of data. Deep learning has also been successfully applied in medical image segmentation, such as prostate segmentation in MR images [20], knee cartilage segmentation [21], ventricular segmentation in ultrasound images [22], and tissue segmentation in breast images [23]. However, there are still many problems in the deep learning method. That is, the information transfer between neurons is one way. In the human visual neural network, in addition to the feedforward connection, there are a large number of feedback connections and lateral connections [24, 25]. Studies have shown that the number of feedback connections is several times the number of feedforward connections [26, 27]. The feedforward neural network can perform certain sensing tasks, but there are still problems with it being sensitive to noise, relying on a large number of samples, poor interpretability, and lack of adaptability and robustness. The feedback neural circuit constitutes an important feedback regulation mechanism. The feedback mechanism plays a very important role in selective attention [28], target location and segmentation [29], feature clustering [30], long-term and short-term memory [31], multitask coordination, and environmental adaptability. Thus, to achieve more advanced intelligence, it is not sufficient to rely solely on a feedforward network with powerful mapping capabilities. For this reason, many scholars began to study the feedback convolutional neural network. For example, Cao proposed a feedback convolutional neural network. In this feedback mechanism, the advanced semantic tag is set as a preamble to infer the activation state of hidden layer neurons. It helps us better visualize and understand the deep work of neural networks and capture the visual attention of intended objects [32]. However, it does not propose a targeted solution to the optimization of the feedback mechanism. Wang et al. proposed a feedback mechanism for convolutional neural networks by constructing a residual attention network. Although the network has improved in object recognition accuracy, there are no corresponding explanation and optimization operation for the feedback mechanism [33]. Amir et al. proposed a network architecture based on general feedback, using existing RNNs for instantiation, and the experimental results are improved compared with the existing feedforward network. But it does not explain the specific logic of the feedback mechanism [34]. At the same time, relevant scholars have proposed a number of convolutional neural networks with feedback mechanisms and applied the network to image classification and image recognition [35–38]. Although these practical applications have achieved certain effects (such as improved accuracy), there are still problems such as unclear feedback mechanism, optimization of feedback mechanism, and computational efficiency. Moreover, there is no corresponding feedback mechanism convolutional neural network model for medical image segmentation. These defects of deep learning theory limit its further development and application in the field of medical image segmentation and have become the technical bottleneck of medical image segmentation technology that affects modern medical diagnosis. Therefore, a feedback convolutional neural network with higher optimization characteristics must be established.

In view of this, this paper analyzes the working mechanism of visual attention in the deep convolutional neural network, uses the target-driven method to perform neuron screening to construct the feedback adjustment mechanism, and then proposes the feedback optimization problem in deep convolutional nerves. In the method of exploring the feedback optimization problem, two feedback optimization algorithms based on greedy strategy are proposed. Through these two different algorithms, a new feedback adjustment mechanism is proposed in the deep convolutional network. This paper refers to this as the feedback adjustment mechanism of the convolutional neural network (FCNN). Finally, the algorithm is used to analyze and summarize the actual medical image segmentation.

Section 2 of this paper mainly explains the deep learning model of the feedback adjustment mechanism.  Section 3 systematically expounds the greedy method proposed in this paper to solve the feedback optimization problem.  Section 4 applies the deep learning model with the feedback mechanism proposed in this paper to the field of medical image segmentation compared with the mainstream segmentation algorithm. Finally, the full paper is summarized and discussed.

## 2. Mathematical Modeling of Feedback Mechanism CNN

### 2.1. Human Vision and Feedback Mechanism

Although the feedforward convolutional neural network has achieved great success in computer vision tasks, it does not have more feedback connections. CNN is a method of simulating human characteristics. However, it has no feedback mechanism. In view of this revelation, this paper considers adding a feedback connection model to the convolutional neural network to make the convolutional neural network more humanized to obtain better application effects.

Through target-driven feedback control, the accuracy of human detection and recognition of targets is improved in complex scenes. It enables the vision system to generate selectivity for neuron responses when processing visual information [39]. In addition, convolutional neural networks have powerful object recognition capabilities [40]. Recent studies have shown that [41, 42] internal neurons of convolutional neural networks for classification purposes can learn to express a variety of visual semantic patterns from massive images, for example, from simple edge features and color features to complex target local features or even complete targets. It shows that the convolutional neural network can segment objects in the image from layers that are simple to complex mode representations.

**Figure 1:**
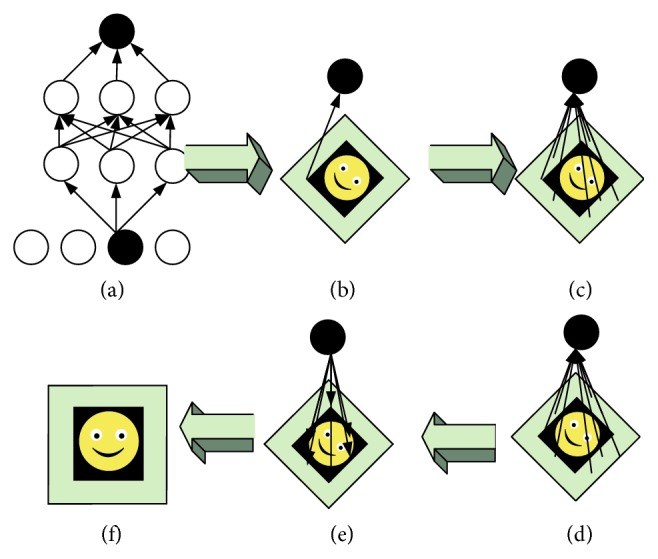
Modeling definition of the feedback mechanism.

Inspired by the above phenomena, this paper can imitate the working mechanism of visual attention in the deep convolutional neural network for classification purposes and perform neuron screening in a target-driven manner to construct a feedback adjustment mechanism. Here, a simple example is given to clarify the feedback-modeling problem mentioned in this article. As shown in Figures 1(a) and 1(b), given an input image, the image has a simple face. Assuming that a convolutional neural network is trained to determine whether there is a human face in the image, the image is sent to the convolutional neural network. In the classification neurons at the highest level of the network, the neurons corresponding to the face category will be highly activated. The neurons here are the target neurons in this paper, denoted as *P*. In this process, there are multiple paths between a pixel in the input image and the target neuron *F*. We abstract all of these pathways into a connecting pathway (CP), which is used to indicate that a pixel is connected to the target neuron. Typically, all pixels within the field of view of the target neuron will be connected to the target neuron. This paper assumes that the target neuron field of view covers the full picture. Therefore, all the pixels and *F* in the figure have their own connection paths, as shown in Figure 1(c). Let *P* be the set of all these pathways. The visual information of the face and the background in the image is transmitted to the target neuron *F* through *P* in a bottom-up manner. In this paper, *R* is used to indicate a rule for determining whether a connected path is connected to a target pixel and a target neuron. Then, the set *P* can be divided into two subsets, *T* and *B*, according to rule *R*. Therefore, the following questions are defined in this paper.

Abstract definition of feedback-modeling problem is as follows: assume *P* = {all pixels to *F*′s CPs}, *T* = {all target pixels to *F*′s CPs}, and *B* = {all nontarget pixels to *F*′s CPs}; find the rules satisfying *R*. Let *P* = *T* ∪ *B* and *T* ∩ *B* = Ф.

### 2.2. Mathematical Modeling of the Feedback Adjustment Mechanism

#### 2.2.1. New Explanation of Deep Neural Networks

Excellent deep convolutional network models are constructed by stacking simple operational layers, including the convolutional layer, the ReLU layer, and the max layer. For each layer, assuming the input is *X*, it is known that it is not the signal or the output of the previous layer. It is assumed that *x* is composed of *C* channels and the length and width are represented by *S* and *T*, that is, *x* ∈ *R*^*S*×*T*×*C*^. The output thereof is assumed to be *y* and is composed of *C*′ channels, and the length and width are *S*′ and *T*′, that is, *y* ∈ *R*^*S*×*T*×*C*^. Therefore, this paper can build the convolutional layer, the ReLU layer, and the largest layer separately.

The convolutional layers are used to extract different features of the input. The convolutional layer consists of *C*′ convolution kernels, each convolution kernel *k* ∈ *R*^*K*×*K*×*C*^, and then the operation of the convolutional layer is described by the following formula:

(1) $$\begin{array}{c} y_{C^{\prime }}=\overset{C}{\underset{c=1}{\sum }}k_{c^{\prime }c}*x_{c},\;\forall c^{\prime }. \end{array}$$

The ReLU layer is mainly used to increase the nonlinearity of the network without affecting the receptive field of the convolutional neurons. Its corresponding input and output function relationship is as follows:

(2) $$\begin{array}{c} y=\max\left(0,x\right). \end{array}$$

The max layer is mainly used to reduce the dimensions of the output vector and to obtain a degree of invariance to ensure that similar structures can achieve the same output. Max acts on the neighborhood *N* of each signal (*i*, *j*), specifically

(3) $$\begin{array}{c} y_{i,j,c}=\underset{u,v\in N}{\max}x_{i}+u,j+v,c,\;\forall i,j,c. \end{array}$$

The selectivity in the feedforward process is to better understand how selectivity works in neural networks. It also models the feedback mechanism. Therefore, this paper needs to reinterpret the role of the ReLU and the largest layer. In this paper, the max( ) operation in equations (2) and (3) can be replaced by a series of binary switches *z* ∈ {0,1}. Therefore, the ReLU and max layers can be represented in the form of *y*=*zοx*. More specifically, the ReLU layer is represented as *y*=*zοx*, in which *o* represents the multiplication of the signal class level, and the max layer is represented as *y*=*z∗x*, in which ^*∗*^ represents the convolution operation and *z* represents the convolution kernel with a value of 0 and 1.

By reinterpreting the ReLU and max layers as gate operations controlled by input *x*, deep convolutional neural networks can be understood as a bottom-up approach to select the useful information for decision-making in the feedforward process by these gate operations. Then, the information that contributes little to the decision is discarded to achieve the final decision. To ensure versatility and generalization, a large amount of information can be filtered through the ReLU and max layers, and thus, a large number of neurons are activated.

#### 2.2.2. Basic Ideas and Mathematical Modeling of the Feedback Adjustment Mechanism

To ensure the versatility and generalization of the model, the deep convolutional neural network opens up almost all gate operations for the input signal and allows as much information as possible to pass through the entire network.

**Figure 2:**
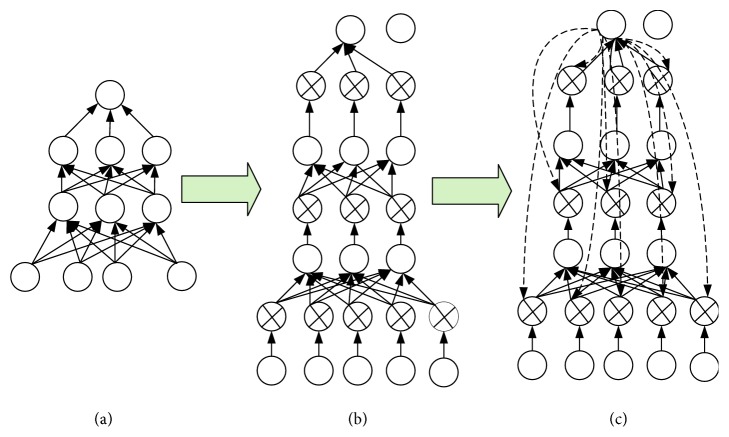
Schematic diagram of the CNN feedback connection constructed in this paper.

In this paper, a binary switch-type hidden variable is introduced for each hidden layer of neurons, which is called a feedback neuron. The control of these new neurons is realized by constructing the feedback connection between the target neurons and all feedback neurons.  Figure 2 shows this process in brief.  Figure 2(a) shows the original CNN, and Figure 2(b) shows the addition of switch-type feedback neurons to each hidden layer neuron.  Figure 2(c) shows the construction of a feedback connection between the target neuron and all feedback neurons.

 
*Bottom-Up.* It inherits the feature selectivity of the ReLU and max layers and passes the image information to the next layer.
 
*Top-Down.* It is implemented by the feedback layer, which passes high-level semantic information to the data layer through gate operations. These gate operations only allow neurons associated with the target to be activated.

The connection path-clipping problem in the previous section is equivalent to the neuron-screening problem under the basic idea and the feedback neuron state control problem. This is given an input signal, and all neurons associated with a given target signal can be successfully screened out from the activated neurons. Then, the connecting pathway formed by these neurons becomes the connecting pathway that we need to screen out. Therefore, this paper needs to further clarify the feedback neuron state control problem.

As mentioned earlier, this paper proposes a large number of switch-type feedback neurons in deep convolutional neural networks. Simultaneously, a simple feedback connection is constructed between the target neuron and these feedback neurons to indicate that the state of the feedback neuron is controlled by the target neuron. By introducing the binary switch *a*, this paper further transforms the feedback mechanism into a numerical optimization problem. Given a signal *I* and a well-learned neural network, the parameter is *w* and a set *Z* ∈ {0,1} of binary switches in the network. This paper assumes that the target neuron output is *S*, and the mapping function of the signal *I* to the target neuron *S* is *f*(*I*, *Z*). This paper attempts to maximize the target output by adjusting the switch state of all feedback layers. The specific description is as follows:

(4) maxZS=fI,Z−λZs.t.zijcl∈0,1,∀i,j,c,ltypel=ReLU or max,

where *z*_*ijc*_^*l*^ represents the binary switch of the position coordinate of the *c*th channel of the *l*th feedback layer being (*i*, *j*). Because our goal is to maximize the target output by activating the fewest neurons, this paper uses the L1 norm to constrain the number of activations of *z*. Thus, this paper applies the mathematical model of the feedback mechanism to the deep convolutional neural network. The problem described in equation (4) is the feedback optimization problem. The solution to the feedback optimization problem is not easy, and it is difficult to obtain the global optimal solution. For the construction of the feedback connection mentioned above, since the purpose of the feedback connection path is to transmit a feedback control signal to the feedback neuron, the feedback neuron works in a predetermined manner. Therefore, this paper can construct a feedback connection in the process of solving the feedback optimization problem. However, from the feedback problem, since it is difficult to obtain the global optimal solution, different solution methods indicate that the calculation method of the feedback control signal is different, which requires different feedback adjustment mechanisms.

## 3. Feedback Optimization Problem by the Greedy Method

### 3.1. Linear Approximation of the Objective Function

CNN is a nonlinear mapping function with a large number of nonlinear mapping layers, for example, the ReLU layer and the max layer. Therefore, *T*_*s*_(*I*) is a function that is highly nonlinear with respect to the input image I. When an input image *I*_0_ is given, Taylor expansion is performed on *T*_*s*_(*I*) near *I*_0_ and *T*_*s*_(*I*) is linearly approximated [42–44]. The result of the first-order Taylor expansion is as follows:

(5) $$\begin{array}{c} T_{s}\left(I\right)\approx T_{s}\left(I_{0}\right)+T_{s}^{\prime }\left(I_{0}\right)\left(I-I_{0}\right). \end{array}$$

**Figure 3:**
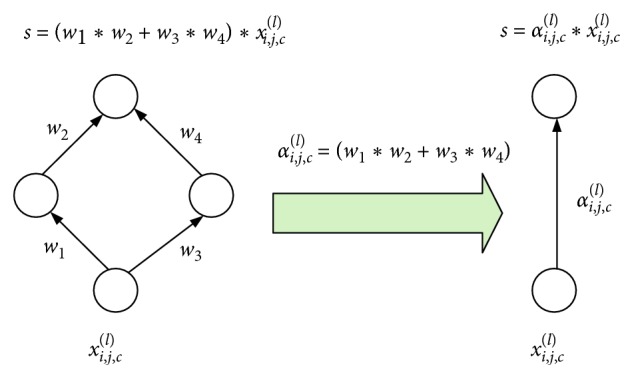
Derivation flow chart of the neuron contribution coefficient.

In this paper, two approximations are used to achieve the approximation of *T*_*s*_(*I*). Specifically, when the input image is known, the state of the neurons inside the network is activated when the network completes the first pretransmission. (1) At this time, this article fixes the door state of the ReLU and max layers. The closed door is always closed, and the open door is fixed to open. The state of the two types of layers is no longer changed. (2) The approximate expression of the remaining nonlinear layers is obtained by Taylor expansion. After completing these operations, *T*_*s*_(*I*) is converted into the output of a linear neural network. Here, the feedback layer is added to each ReLU layer, and the target function is updated into a linear nested function. It is assumed that *T*_*s*_^*∗*^(*I*, *Z*), *T*_*s*_^*∗*^(*I*, *Z*) can be expressed by any feedback layer to form a linear combination of functions, specifically

(6) $$\begin{array}{c} T_{s}^{*}\left(I,Z\right)=\underset{ijc}{\sum }\alpha_{ijc}^{l}z_{ijc}^{l}x_{ijc}^{l}, \end{array}$$

where *x*_*ijc*_^*l*^ is the input of the feedback neuron at (*i*, *j*) above the channel *c* of the feedback layer *l*, *z*_*ijc*_^*l*^ represents the feedback neuron at the corresponding position, and *α*_*ijc*_^*l*^ is represented as the contribution weight (CW). *α*_*ijc*_^*l*^ is determined by the neural connection pathway between the feedback neuron and the target neuron *T*_*s*_. The flow of the neuron contribution coefficient is shown in Figure 3. It can be seen from Figure 3 that, in the linear neural network, it is assumed that there are two paths between the target neuron *T* and a certain neuron *x*_*ijc*_^*l*^ in the middle layer, and each path has its own corresponding weight, such as *w*_1_, *w*_2_, *w*_3_, and *w*_4_. In this paper, *α*_*ijc*_^*l*^ can be obtained by the weight on the path in Figure 3 so that the target neuron *T* and the neuron *x*_*ijc*_^*l*^ can be abstracted into a connection path with a weight of *α*_*ijc*_^*l*^.

In this paper, we obtain the linear approximation of the objective function in the feedback optimization problem by formula (6). To further simplify the problem, this paper abandons the regularization requirement of *Z* so that all *Z*'s that contribute to the objective function are opened. At this time, the feedback optimization problem is transformed into the following problem:

(7) maxZTs∗I,Zs.t.zijcl∈0,1, ∀l,i,j,c, typel=ReLU.

Formula (7) has no constant term because *x*_*ijc*_^*l*^ in *T*_*s*_^*∗*^(*I*, *Z*) is the output of the ReLU neuron. The constant term is calculated in *x*_*ijc*_^*l*^.

### 3.2. Feedback Optimization Problem by the Greedy Method

#### 3.2.1. Feedback Recovery Algorithm

This paper proposes a top-down layer-by-layer optimization method to update the feedback neurons *Z* of each layer to obtain the maximum objective function *T*. For a particular feedback layer 1, input *x*_*ijc*_^*l*^ is given to determine some type of visual pattern in the image space, and the contribution coefficient *α*_*ijc*_^*l*^ is used to express the contribution of the visual mode to the target neuron. Therefore, in this paper, the positive contribution of *x*_*ijc*_^*l*^ can be retained in a top-down order, while the negative contribution value of *x*_*ijc*_^*l*^ is eliminated, thereby maximizing the target neuron *T*. Specifically, at a certain layer, the state of the switch *z*_*ijc*_^*l*^ is updated according to the symbol *α*_*ijc*_^*l*^, and the neuron contributing to the negative value is turned off. At this time, the neural network of the layer to the target neuron is roughly cropped. Then, *x*_*ijc*_^*l*^ retained in the layer is expanded to the next layer, and the contribution coefficient of each neuron in the next layer is recalculated in the new network structure and processed according to the same strategy. In this paper, such a strategy is applied to each feedback layer in a top-down manner and iterates until convergence. This algorithm is called the feedback recovery algorithm. In this algorithm, it is assumed that the neural network has a total of *N* feedback layers, and we record the target function after updating the feedback layer *l* as *T*_*l*_. For the convenience of description, subscript *k* is used instead of *i*, *j*, and *c*. The mathematical proof process of the algorithm is given below.

To prove that the feedback recovery algorithm can make the feedback optimization problem obtain the local optimal solution, this paper needs to prove that the objective function *T* will increase the value of the objective function after each iteration; that is, this paper needs to prove *T*_*N*_ ≤ *T*_1_. We can use mathematical induction to complete the proof. To this end, this paper first proves that *T* ≤ *T*_*N*_ and *T*_*N*_ ≤ *T*_*N*−1_ and proves that *T*_*l*_ ≤ *T*_*l−*1_ under the assumption that *T*_*l+*1_ ≤ *T*_*l*_ is established.

(1) Assumption *l* *=* *N*

This paper expands *T* through the *N*th feedback layer, namely,

(8) $$\begin{array}{c} T=\underset{k}{\sum }\alpha_{k}^{\left(N\right)}z_{k}^{\left(N\right)}x_{k}^{\left(N\right)}, \end{array}$$

where *x*_*k*_^(*N*)^ is the *N*th ReLU layer output neuron; thus, *x*_*k*_^(*N*)^ ≥ 0. Available from *z*_*k*_^(*N*)^ ∈ {0,1},

(9) $$\begin{array}{c} \alpha_{k}^{\left(N\right)}z_{k}^{\left(N\right)}x_{k}^{\left(N\right)}\leq \alpha_{k}^{\left(N\right)}\delta \left(\alpha_{k}^{\left(N\right)}\right)x_{k}^{\left(N\right)}. \end{array}$$

Let *z*_*k*_^(*N*)^⟶*z*_*k*_^′(*N*)^=*δ*(*α*_*k*_^(*N*)^) and *α*_*k*_^′(*N*)^=*α*_*k*_^(*N*)^*∗z*_*k*_^′(*N*)^ ≥ 0, and then

(10) $$\begin{array}{c} T\leq T_{N}=\underset{k}{\sum }\alpha_{k}^{\prime \left(N\right)}x_{k}^{\left(N\right)}. \end{array}$$

After updating all *z*_*k*_^(*N*)^ of the *N*th feedback layer, *T*_*N*_ can be expressed by the *N* − 1th feedback layer. Here, *α*_*k*_^(*N* − 1)^depends on *α*_*k*_^(*N*)^. Therefore, when *α*_*k*_^(*N*)^ is adjusted, *α*_*k*_^(*N* − 1)^ is also updated to ${\hat{\alpha }}_{k}^{\left(N-1\right)}$; thus,

(11) $$\begin{array}{c} T_{N}=\underset{k}{\sum }{\hat{\alpha }}_{k}^{\left(N-1\right)}z_{k}^{\left(N-1\right)}x_{k}^{\left(N-1\right)}. \end{array}$$

Then, this paper uses the above method to update *z*_*k*_^(*N* − 1)^ and ${\hat{\alpha }}_{k}^{\left(N-1\right)}$ to obtain *T*_*N*−1_:

(12) $$\begin{array}{c} T_{N}\leq T_{N-1}. \end{array}$$

(2) Assumption *T*_*l*+1_ ≤ *T*_*l*_

Fix *z*_*k*_^(*N*)^, *z*_*k*_^(*N* − 1)^,   …  , *z*_*k*_^(*l*+1)^, and then

(13) $$\begin{array}{c} T_{l}=\underset{k}{\sum }\alpha_{k}^{\prime \left(l\right)}x_{k}^{\left(l\right)}. \end{array}$$

*x*_*k*_^(*l*)^ can be represented by *x*_*k*′_^(*l* − 1)^ together with the convolutional layer weight *ω*_*k*′_^(*l* − 1)^:

(14) xkl=ReLU∑k′ωk′l−1zk′l−1xk′l−1.

If *ω*_*k*′_^(*l* − 1)^*z*_*k*′_^(*l* − 1)^*x*_*k*′_^(*l* − 1)^ < 0, then *T*_*l*_ is equivalent to one or more 0 items, which can be ignored. Therefore, this article only needs to focus on the situation when ∑_*k*′_*ω*_*k*′_^(*l* − 1)^*z*_*k*′_^(*l* − 1)^*x*_*k*′_^(*l* − 1)^ ≥ 0, and then

(15) $$\begin{array}{c} x_{k}^{\left(l\right)}=\underset{k^{\prime }}{\sum }\omega_{k^{\prime }}^{\left(l-1\right)}z_{k^{\prime }}^{\left(l-1\right)}x_{k^{\prime }}^{\left(l-1\right)}. \end{array}$$

Therefore,

(16) $$\begin{array}{c} S_{l}=\underset{k}{\sum }\alpha_{k}^{\prime \left(l\right)}\underset{k^{\prime }}{\sum }\left(\omega_{k^{\prime }}^{\left(l-1\right)}z_{k^{\prime }}^{\left(l-1\right)}x_{k^{\prime }}^{\left(l-1\right)}\right). \end{array}$$

Since *α*_*k*_^′(*l*)^ ≥ 0,

(17) $$\begin{array}{c} S_{l}=\underset{k^{\prime }}{\sum }\left(\underset{k^{\prime }}{\sum }\left(\alpha_{k}^{\prime \left(l\right)}\omega_{k^{\prime }}^{\left(l-1\right)}\right)z_{k^{\prime }}^{\left(l-1\right)}x_{k^{\prime }}^{\left(l-1\right)}\right). \end{array}$$

Update the control gate state of the *l* − 1th feedback layer on the basis of *S*_*l*_*l* so that

(18) $$\begin{array}{c} \alpha_{k^{\prime }}^{\left(l-1\right)}=\frac{\partial S_{l}}{\partial x_{k^{\prime }}^{\left(l-1\right)}}\left(\underset{k^{\prime }}{\sum }\alpha_{k}^{\prime \left(l\right)}\omega_{k^{\prime }}^{\left(l-1\right)}\right)z_{k^{\prime }}^{\left(l-1\right)}. \end{array}$$

It is available by updating *z*_*k*′_^(*l* − 1)^⟶*z*_*k*′_^′(*l* − 1)^ as follows:

(19) $$\begin{array}{c} z_{k^{\prime }}^{\prime \left(l-1\right)}=\delta \left(\frac{\partial S_{l}}{\partial x_{k^{\prime }}^{\left(l-1\right)}}\right). \end{array}$$

It is available by updating *α*_*k*′_^(*l* − 1)^⟶*α*_*k*′_^′(*l* − 1)^ as follows:

(20) $$\begin{array}{c} \alpha_{k^{\prime }}^{\prime \left(l-1\right)}=\left(\sum_{k^{\prime }}\alpha_{k}^{\prime \left(l\right)}\omega_{k^{\prime }}^{\left(l-1\right)}\right)*\delta \left(\sum_{k}\alpha_{k}^{\prime \left(l\right)}\omega_{k^{\prime }}^{\left(l-1\right)}\right)\\ =\frac{\partial S_{l}}{\partial x_{k^{\prime }}^{\left(l-1\right)}}*\delta \left(\frac{\partial S_{l}}{\partial x_{k^{\prime }}^{\left(l-1\right)}}\right)\\ =\left(\sum_{k}\alpha_{k}^{\prime \left(l\right)}\omega_{k^{\prime }}^{\left(l-1\right)}\right)*z_{k^{\prime }}^{\prime \left(l-1\right)}\\ \geq \left(\sum_{k}\alpha_{k}^{\prime \left(l\right)}\omega_{k^{\prime }}^{\left(l-1\right)}\right)*z_{k^{\prime }}^{\left(l-1\right)}. \end{array}$$

Since *α*_*k*′_^(*l* − 1)^ ≥ 0 and *x*_*k*′_^(*l* − 1)^ ≥ 0,

(21) $$\begin{array}{c} S_{l-1}=\underset{k^{\prime }}{\sum }\alpha_{k^{\prime }}^{\left(l-1\right)}x_{k^{\prime }}^{\left(l-1\right)}\\ \geq \underset{k}{\sum }\left(\underset{k^{\prime }}{\sum }\alpha_{k^{\prime }}^{\prime \left(l\right)}\omega_{k^{\prime }}^{\left(l-1\right)}\right)z_{k^{\prime }}^{\left(l-1\right)}x_{k^{\prime }}^{\left(l-1\right)}=S_{l}. \end{array}$$

That is,

(22) $$\begin{array}{c} S_{l}\leq S_{l-1}. \end{array}$$

In summary, after the first iteration is completed,

(23) $$\begin{array}{c} S_{N}\leq S_{1}. \end{array}$$

Because the number of neurons given in this paper is limited, each iteration performs a cropping operation, so the value of the objective function *T* will continue to increase until it converges.

To qualitatively understand the feedback effect of FR, this paper combines the FR algorithm with the commonly used deep convolutional neural network framework VGGNet [45]. VGGNet pretrained the object classification task on the Imagenet2012 dataset. The visualization is generated after the network is cropped using the FR algorithm. The visualization and energy diagrams are explained in detail here.

*(1) Visualization Map and Energy Map*. When the FR algorithm converges, the gradient of the target neuron is set to 1, and the gradient backtransfer calculation is started from the target neuron. Finally, a gradient map is obtained in the image space. The gradient map is also 3-channel, which is consistent with the input image size. To visualize the gradient map, the normalization process is performed by a min-max method with a constant, specifically (255*∗*(*x* − min/max − min)), which in turn forms a visualization map. At the same time, to measure the target correlation of each pixel, the sum of the absolute values of the three channels of the gradient map at each position is calculated, and then the energy map is normalized by the L2 norm.

#### 3.2.2. Feedback Selective Algorithm

**Figure 4:**
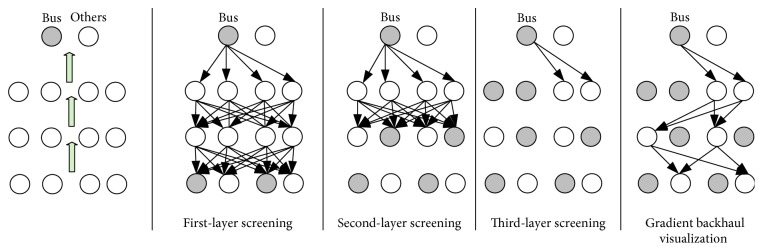
Schematic diagram of the feedback selective algorithm process.

Because the FR algorithm constantly adjusts the contribution coefficient of each neuron in the optimization process, the FR algorithm loses the neuron-screening ability, which inspires us to adjust the contribution coefficient as much as possible in the optimization process. In this section, this paper proposes another optimization method that updates the feedback neuron state of each layer without changing the contribution coefficients during an iterative process. To achieve this goal, this article optimizes the objective function in a bottom-up manner. The objective function *T* is optimized by means of layer adjustment. The process of one iteration of the FS algorithm is shown in Figure 4. Given an input image containing an aircraft, this article uses the category of neurons corresponding to “bus” as the target neuron and then optimizes each feedback layer layer-by-layer in a bottom-up manner, repeating this process until convergence. Once the optimization is complete, it is equivalent to completing the selective filtering of the network. Finally, a visual map and an energy map are obtained by a gradient from the target neuron to the image input space.

The proof of convergence of the FS algorithm is given below. To prove that the FS algorithm can be a feedback optimization problem to obtain a local optimal solution, it is also proven by mathematical induction. In this case, this article needs to prove that *T*_1_ ≤ *T*_*N*_. Thus, it is first necessary to prove that *T* ≤ *T*_1_ and *T*_1_ ≤ *T*_2_, and then the condition *T*_*l*−_ ≤ *T*_*l*+1_ can be proven on the premise that *T*_*l*−1_≤*T*_*l*−_ is assumed.

(1) *l* = 1:
This paper expands *T* through the first feedback layer, namely,
  (24) $$\begin{array}{c} T=\sum_{k}\alpha_{k}^{\left(1\right)}z_{k}^{\left(1\right)}x_{k}^{\left(1\right)}, \end{array}$$
  where *x*_*k*_^(1)^ represents that the first ReLU layer is an output neuron, so *x*_*k*_^(1)^ ≥ 0.

Available from *z*_*k*_^(1)^ ∈ {0,1},

(25) $$\begin{array}{c} \alpha_{k}^{\left(1\right)}z_{k}^{\left(1\right)}x_{k}^{\left(1\right)}\leq \alpha_{k}^{\left(1\right)}\delta \left(\alpha_{k}^{\left(1\right)}\right)x_{k}^{\left(1\right)}. \end{array}$$

Let *z*_*k*_^(1)^⟶*z*_*k*_^′(1)^=*δ*(*α*_*k*_^(1)^), then

(26) $$\begin{array}{c} x_{k}^{\prime \left(1\right)}=x_{k}^{\left(1\right)}*\delta \left(\alpha_{k}^{\prime \left(1\right)}\right)\geq 0,\\ T\leq T_{1}=\sum_{k}\alpha_{k}^{\left(1\right)}x_{k}^{\prime \left(1\right)}. \end{array}$$

After updating all *z*_*k*_^(1)^ of the first feedback layer, *T*_1_ can be expressed by the second feedback layer. Here, *x*_*k*_^(2)^ depends on *α*_*k*_^(1)^. Therefore, when *α*_*k*_^(1)^ is adjusted, *α*_*k*_^(2)^ will also be updated to ${\hat{\alpha }}_{k}^{\left(2\right)}$, so

(27) $$\begin{array}{c} T_{1}=\sum_{k}\alpha_{k}^{\left(2\right)}z_{k}^{\left(2\right)}{\hat{x}}_{k}^{\left(2\right)}. \end{array}$$

Then, this paper uses the above method to update *z*_*k*_^(2)^ and ${\hat{x}}_{k}^{\left(2\right)}$ to obtain *T*_2_, so

(28) $$\begin{array}{c} T_{1}\leq T_{2}. \end{array}$$

(2) Assumption *T*_*l*−1_ ≤ *T*_*l*_:
  Fix *z*_*k*_^(2)^, *z*_*k*_^(2)^,…, *z*_*k*_^(*l* − 1)^, then

(29) $$\begin{array}{c} T_{l}=\underset{k}{\sum }\alpha_{k}^{\left(l\right)}x_{k}^{\prime \left(l\right)}. \end{array}$$

*S*_*l*_ can also be represented by *x*_*k*_^(*l*+1)^, so

(30) $$\begin{array}{c} T_{l}=\underset{k}{\sum }\alpha_{k}^{\left(l+1\right)}{\hat{x}}_{k}^{\prime \left(l+1\right)}z_{k}^{\left(l+1\right)}. \end{array}$$

Among them,

(31) x^kl+1=ReLU∑k′ωk′lzk′lxk′′l≥0.

If *ω*_*k*′_^(*l* − 1)^*z*_*k*′_^(*l* − 1)^*x*_*k*′_^(*l* − 1)^ < 0, then *T*_*l*_ is equivalent to one or more 0 items, which can be ignored. Therefore, this article only needs to focus on the situation when ∑_*k*′_*ω*_*k*′_^(*l* − 1)^*z*_*k*′_^(*l* − 1)^*x*_*k*′_^(*l* − 1)^ ≥ 0, and then

(32) $$\begin{array}{c} x_{k}^{\left(l\right)}=\underset{k^{\prime }}{\sum }\omega_{k^{\prime }}^{\left(l-1\right)}z_{k^{\prime }}^{\left(l-1\right)}x_{k^{\prime }}^{\left(l-1\right)}. \end{array}$$

Therefore,

(33) $$\begin{array}{c} S_{l}=\underset{k}{\sum }\alpha_{k}^{\left(l+1\right)}{\hat{x}}_{k}^{\left(l+1\right)}z_{k}^{\left(l+1\right)}\\ \leq \underset{k}{\sum }\alpha_{k}^{\left(l+1\right)}\delta \left(\alpha_{k}^{\left(l+1\right)}\right){\hat{x}}_{k}^{\left(l+1\right)}\\ =\underset{k}{\sum }\alpha_{k}^{\left(l+1\right)}x_{k}^{\prime \left(l+1\right)}=S_{l+1}. \end{array}$$

That is, *S*_*l*_ ≤ *S*_*l*+1_.

Update $\hat{x}{}_{k}^{(l+1)}⟶x_{k}^{(l+1)}\text{ and }$*z*_*k*_^(*l*+1)^⟶*z*_*k*_^′(*l*+1)^ by the following formula:

(34) $$\begin{array}{c} x_{k}^{\prime \left(l+1\right)}={\hat{x}}_{k}^{\left(l+1\right)}*\delta \left(\alpha_{k}^{\left(l+1\right)}\right),\\ z_{k}^{\prime \left(l+1\right)}=\delta \left(\alpha_{k}^{\left(l+1\right)}\right). \end{array}$$

Based on mathematical induction, after the first iteration, the following can be obtained:

(35) $$\begin{array}{c} S_{l}\leq S_{N}. \end{array}$$

Because the number of neurons in this paper is limited, each iteration performs a selection operation, so the value of the objective function *T* continues to increase. It will eventually converge.

## 4. Medical Image Segmentation Based on FCNN

### 4.1. Medical Image Segmentation Design

**Figure 5:**
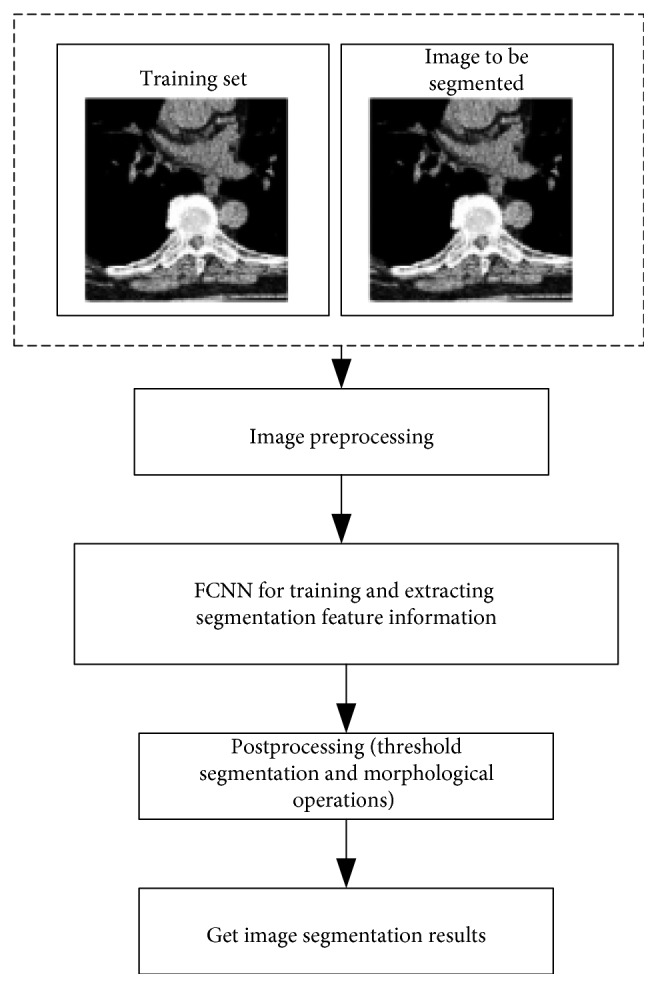
Medical image segmentation method based on FCNN.

In this paper, a convolutional neural network with a feedback mechanism is constructed. First, fixed-size image block samples are extracted from the trained image set that has been preprocessed. Feature learning is performed through unlabeled image block samples, and the initial parameters of each layer of the network are trained. Then, further fine-tuning through the labeled image block samples is performed so that the convolutional neural network has a classification function. Then, the image block samples to be segmented are classified, and the part of the content to be marked is added to the black and white binary image as the initial segmentation result. Finally, the results of threshold segmentation and morphological processing are used to optimize the results of accurate segmentation of certain medical images. The technical details of the medical image segmentation method in this paper are shown in Figure 5.

### 4.2. Medical Image Segmentation Process Based on FCNN

First, the original sample data are generated. The ultimate goal of constructing the deep neural network in this paper is to classify the image pixels and then use the classification results to achieve medical image segmentation, taking into account the neighboring relationship of similar pixels in the segmentation task. Therefore, an image block with a target pixel point of 25 × 25 is used as the sample to be tested, and the category of the central pixel is determined according to the classification result of the image block. To standardize the data and eliminate the influence of the dimensions, it is necessary to first normalize the image, and the gray value range of the image block is limited to 0∼1. The formula is as follows:

(36) $$\begin{array}{c} f^{\prime }\left(x,y\right)=\frac{f\left(x,y\right)-\min}{\max-\min}, \end{array}$$

where max and min represent the maximum and minimum values, respectively, in the gray values in the image.

To obtain a robust learning network, it is necessary to add a certain degree of noise pollution to the original sample data and then use the contaminated data as an input to the first-layer learning network. The first-layer network is trained to minimize the error between the reconstructed output and the uncontaminated raw data.

Using the trained parameters, the output of the hidden layer (middle layer) in the first-layer network is calculated and trained as the input of the second layer, and the second-layer network is generated by using the newly obtained parameters. Then, the output of the hidden layer in the second-layer network is used as the input to the third layer. In this iterative training process, the feedback mechanism is constructed by using the third section, and finally, the convolutional neural network with the feedback mechanism is obtained. In this process, the hidden layer output in each layer of the network is the “depth feature” of the image. To ensure that the deep neural network has the classification function, it is necessary to use supervised learning to fine tune the whole network to ensure that the features correspond to the categories. The specific method constructs a complete feedback mechanism convolutional neural network by using the parameters of each layer of the previous network and adds an output layer at the end of the whole network to construct a feedforward deep neural network. Then, the output result is compared with the true value of the data, and the network parameters are adjusted according to the difference between them so that the input sample can output its corresponding category after a series of network mapping, thus ensuring the ability to classify the sample.

### 4.3. Image Segmentation Result Optimization

After obtaining the classification result of the image block, the corresponding central pixel point is mapped into the appropriate category according to the category label of each image block, thereby obtaining an initial segmentation result. However, due to the use of the gray level of the image block as the classification basis, it may be missegmented. To eliminate the phenomenon of missegmentation, the initial segmentation results are initially processed by threshold segmentation; the threshold is set according to the grayscale distribution of all pixels classified into a certain feature region, and then the pixel points that do not obviously belong to the tumor tissue are deleted.

To obtain a better segmentation effect, this paper uses open and closed operations in the morphological processing method to optimize the segmentation results [46]. The main function of the open operation is to eliminate the bulging edges of the image and the isolated spots. The main function of the closing operation is to fill the gaps and the concaves inside the image. In this paper, to optimize the segmentation results, the above two morphological operations will be used in combination.

## 5. Experimental Analysis

### 5.1. Tumor Image Segmentation Experiment

In this experiment, to verify the validity and robustness of the proposed method, the image segmentation algorithm proposed in this paper is used to segment the medical image. The medical image is segmented by other methods, and finally, a performance comparison table of these algorithms is given. In this paper, the Dice ratio algorithm is used to evaluate the accuracy of the segmentation result, which indicates the similarity between the experimental segmentation result and the expert manual segmentation gold standard. The images of the MRI modes used in this paper are from the BRATS [47–50] contest, which contains the four modes T1, T1c, T2, and FLAIR. The training data contain 30 patients' real datasets and 50 simulated patient datasets. In this paper, 70% of the data are used as training data, 30% of the data are used as test data, and a cross-validation method is used to obtain the segmentation results. All training data are standard data, which have been segmented by professionals in advance, wherein pixel values 1, 2, 3, and 4 represent necrotic tissue, edema tissue, nonenhanced tumor, and enhanced tumor, respectively, and 0 represents normal tissue.

The comparison between the method proposed in this paper and other methods in the BRATS [49, 50] contest is shown in Table 1. This paper selects the best performing Zhao method (the Monte Carlo random-based supervoxel clustering method), the Baner method, the ordinary Menze algorithm, and the CNN method as the comparison objects.

Table 1 shows that the correct rate for the convolutional neural network segmentation method proposed is as high as 85.9%. This method is not only superior to the general tumor image segmentation method for tumor segmentation and recognition accuracy but also 4.2% better than the current (Zhao) algorithm. At the same time, the stability of the method is superior to that of the Zhao method (the method variance is 0.08, and the variance of the Zhao method is 0.09). In addition, the feedback mechanism convolutional neural network proposed in this paper is also superior to the general CNN method (the accuracy of the traditional CNN method is 80.2%). It can be seen from this that although the traditional CNN segmentation method is less effective than the proposed method, the CNN method can also achieve the accuracy of Zhao's proposed algorithm (the accuracy of the CNN method is 80.2%, and the accuracy of the Zhao method is 81.7%), which fully demonstrates the great advantages of deep learning theory in medical image segmentation.

### 5.2. Spine Image Segmentation Experiment

Spinal CT images for deep learning are derived from two datasets from SpineWeb [51], one of which contains five body data that are labeled to segment only the vertebral body without the transverse processes, spinous processes, and pedicles. The image resolution is 1.0 × 1.0 × 1.0 mm^3^, the scan matrix size is 512 × 512, the number of slice images is between 30 and 88, and the other dataset contains 20 individual data. The segmented complete vertebral body has a resolution of 0.35 × 0.35 × 1 mm^3^, the acquired slice image size is 512 × 512, and the number of sliced images is 255 to 950.

In the dataset of 5 individuals' data, the numbers of network training, testing, and verification are 3, 1, and 1, and the dataset containing 20 individuals retains one data for verification. All other data are used for network training and cross-testing, and the ratio is 8 : 2. Since the black background area is large and unevenly distributed in the image of the dataset of 5 individuals, the data are preprocessed, and the training dataset is cropped to 128 × 128 without reducing the image resolution. The image is normalized for each batch-read image, and the specific processing method is shown in formula (36). There are insufficient annotated images for network training. Therefore, it is necessary to expand the original training dataset. In each training iteration, the input training image is elastically deformed by the density deformation field obtained using the 3 × 3 grid control points and fractal interpolation. A new variant of the training dataset is derived, which is mainly used to verify the validity and reliability of the data expansion method.

**Figure 6:**
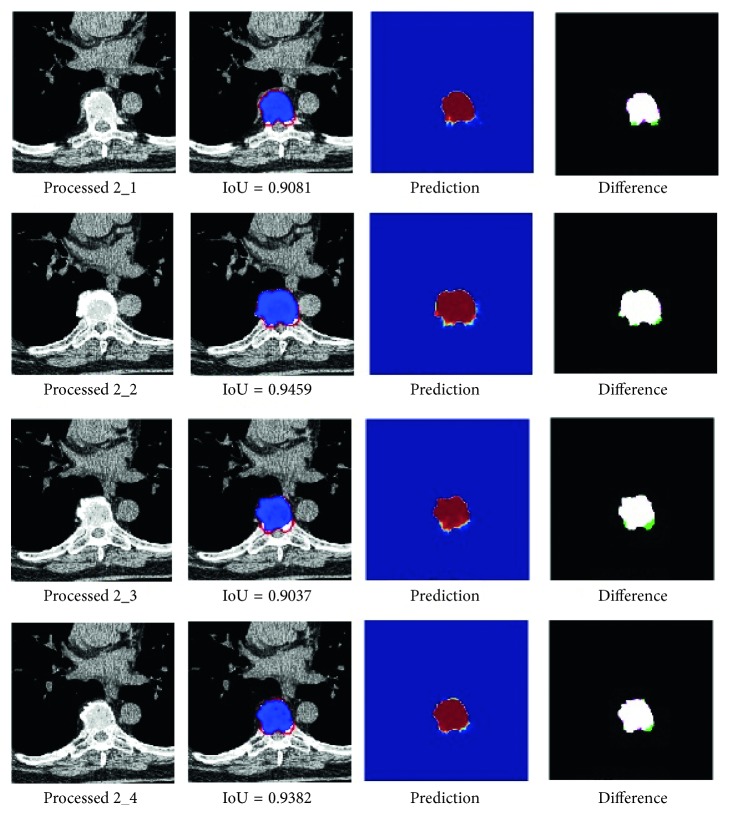
CT image segmentation results of Dataset 4.

Figure 6 shows segmentation results of a set of verification experiments that is loaded into a small dataset-training model and is randomly selected. The network prediction results are shown in Figure 6, along with the results of the segmentation and the standard overlapping of the combined results (IoU) and the difference between them. The red outline in the second column in Figure 6 is the standard outline drawn by hand, the blue area is the prediction result, and the third column is the algorithm division result. In the fourth-column difference map, the white area represents the coincident area, the pink area is undersegmented, and the green area represents the overdivided area. The trained network model was used to analyze the volume of data that were not involved in training and testing. It contained 58 slice images, the average IoU was 0.8037, and the average Dice value was 0.8579. From the analysis of the results of the verification output, the data expansion method has a good effect, and to some extent, it can compensate for the lack of training data.

## 6. Conclusion

Inspired by the visual attention mechanism in the human visual system, this paper presents the problem definition of the feedback adjustment mechanism in the deep convolutional neural network with object classification as the task. The essential goal of feedback is to target neuron screening in a target-driven manner. From the perspective of feedback, the composition of the convolutional neural network is reinterpreted, and the mechanism of stimulus-driven neuron screening and its problems in the feedforward process of convolutional neural networks are noted. Then, the paper introduces the feedback neuron and the feedback layer with the goal of maximizing the target neuron output, constructs the mathematical model of the whole feedback mechanism, and clarifies the feedback optimization problem. On this basis, this paper proposes a feedback optimization problem based on the greedy method. Two solving algorithms are also given: a feedback selective (FS) algorithm and a feedback recovery (FR) algorithm. This paper proposes a new framework for the FCNN method based on the FR and FS algorithms. It can effectively capture high-level semantic concepts and project them back into the image space to generate various energy maps with great practical value. The feedback convolutional neural network has the ability to locate and segment the target objects from the image.

At the same time, because it is difficult to find and extract effective features based on medical image segmentation, combined with the feedback convolutional neural network method presented in this paper, a medical image segmentation algorithm based on feedback mechanism convolutional neural network is proposed. To verify the reliability and advantages of the medical image segmentation algorithm proposed in this paper, this method is used to segment the tumor image and the spine image and compare it with the excellent algorithms in the CNN method and BRATS contest. The experimental results show that the proposed method can not only segment the tumor image more accurately but also segment the spine image. The recognition effect is not only superior to the CNN method but also superior to the excellent algorithm of the BRATS competition.

## Figures and Tables

**Table 1:** Comparison of segmentation results of different segmentation methods.

Method type	Accuracy (%)	Variance
Menze	55.1	0.06
Bauer	79.4	0.04
Zhao	81.7	0.09
CNN	80.2	0.09
Proposed method	85.9	0.08

## Data Availability

The data used to support the findings of this study are available from the corresponding author upon request.
